# Identification, Expression Profiling and Fluorescence-Based Binding Assays of a Chemosensory Protein Gene from the Western Flower Thrips, *Frankliniella occidentalis*

**DOI:** 10.1371/journal.pone.0117726

**Published:** 2015-01-30

**Authors:** Zhi-Ke Zhang, Zhong-Ren Lei

**Affiliations:** 1 State Key Laboratory for Biology of Plant Diseases and Insect Pests, Institute of Plant Protection, Chinese Academy of Agricultural Sciences, Beijing, China; 2 Institute of Plant Protection, Ningxia Academy of Agriculture and Forestry Sciences, Yinchuan, China; Russian Academy of Sciences, Institute for Biological Instrumentation, RUSSIAN FEDERATION

## Abstract

Using RT-PCR and RACE-PCR strategies, we cloned and identified a new chemosensory protein (FoccCSP) from the Western flower thrips, *Frankliniella occidentalis*, a species for which no chemosensory protein (CSP) has yet been identified. The FoccCSP gene contains a 387 bp open-reading frame encoding a putative protein of 128 amino acids with a molecular weight of 14.51 kDa and an isoelectric point of 5.41. The deduced amino acid sequence contains a putative signal peptide of 19 amino acid residues at the N-terminus, as well as the typical four—cysteine signature found in other insect CSPs. As FoccCSP is from a different order of insect than other known CSPs, the GenBank FoccCSP homolog showed only 31-50% sequence identity with them. A neighbor-joining tree was constructed and revealed that FoccCSP is in a group with CSPs from Homopteran insects (e.g., AgosCSP4, AgosCSP10, ApisCSP, and NlugCSP9), suggesting that these genes likely developed from a common ancestral gene. The FoccCSP gene expression profile of different tissues and development stages was measured by quantitative real-time PCR. The results of this analysis revealed this gene is predominantly expressed in the antennae and also highly expressed in the first instar nymph, suggesting a function for FoccCSP in olfactory reception and in particular life activities during the first instar nymph stage. We expressed recombinant FoccCSP protein in a prokaryotic expression system and purified FoccCSP protein by affinity chromatography using a Ni-NTA-Sepharose column. Using N-phenyl-1-naphthylamine (1-NPN) as a fluorescent probe in fluorescence-based competitive binding assay, we determined the binding affinities of 19 volatile substances for FoccCSP protein. This analysis revealed that anisic aldehyde, geraniol and methyl salicylate have high binding affinities for FoccCSP, with K_D_ values of 10.50, 15.35 and 35.24 μM, respectively. Thus, our study indicates that FoccCSP may play an important role in regulating the development of the first instar nymph and mediate *F. occidentalis host recognition.*

## Introduction

Olfactory and gustatory chemoreception is crucial for the phytophagous insect selection of host plants [1]. Among these systems the insect olfaction system is particularly sensitive and sophisticated [2–3]. Insects use olfaction to perceive semiochemicals, such as insect pheromones and plant volatiles, for locating food sources, courtship, mating and oviposition sites [4–13]. Within the insect olfaction system, chemosensory proteins (CSPs), such as odorant binding proteins (OBPs), olfactory receptors (ORs) and odorant degrading enzymes (ODEs), are essential. CSPs comprise a family of acidic, low-molecular-mass and soluble olfactory proteins within the chemosensory lymph of insect olfactory receptors and likely play important roles in insect chemoreception, such as recognizing, capturing and transporting hydrophobic chemicals from the environment to olfactory receptors [5, 14–18]. Subsequent studies used a fluorescence-based competitive binding assay to confirm that CSPs are capable of binding a range of chemicals [3, 19–20]. Furthermore, CSPs likely have other functions, such as regulating insect development [21–22] and circadian cycles [23], as well as participating in the immune response [24–25]. Moreover, CSPs have only been found in insects to date [26]. CSPs may have other unclear physiological roles and should be examined in detail.

The first CSP member was identified *by* subtractive hybridization as being expressed in the antennae of *Drosophila melanogaster* and named olfactory specific-D (OS-D) [27] or A-10 [28] for differing from OBPs and was subsequently named sensory appendage proteins (SAPs) [29]. Until 1999, this class of proteins was formally named CSPs for their expression in chemosensory organs (antennae, labrum and tarsi) of the desert locust *Schistocerca gregaria* [30].

Currently, many CSPs have been discovered and identified in several insect orders. Among them, a large number of CSPs have been reported in Lepidoptera [1, 9, 18, 31–35] and Hymenoptera [2, 17, 36–38], and some CSPs have been reported in other orders, such as Blattaria [39–40], Phasmatodea [41–42], Orthoptera [30, 43–44], Diptera [26, 45] and Hemiptera [46].

CSPs are distinct from OBPs. CSPs share no sequence similarity with OBPs [17–18], and whereas OBPs are primarily express in antennae, CSPs are expressed in various insect tissues, including antennae [16, 35, 47], head [3], legs [39–48], thorax [3], wings [43, 49], proboscis [33], pheromone glands [35], maxillary palps [31], ejaculatory duct [25, 51], labial palps [44] and the sub-cuticular epithelium [45]. CSPs have some common characteristics, such as a low molecular mass (10–15 kDa), four highly conserved cysteine residues, six α-helices connected by α-α loops, a wide distribution and a high amino acid identity.

The western flower thrips, *Frankliniella occidentalis* (frankliniella occidentalisfrankliniella occidentalisfrankliniella occidentalishttp://baike.so.com/doc/6150715.htmlThysanoptera, Thripidae), also called alfalfa thrips, was discovered in Hawaii in 1955, first reported in China in 2003 [52], and is an invasive species. Since the 1980s, *F*. *occidentalis* has become a dominant thrips species and gradually spread to America, Europe, Asia, Africa, and Oceania dominant population dominant population to become a worldwide pest [53–55]. *F*. *occidentalis* are frankliniella occidentalisare plant-feeding insects and can cause severe damage to many vegetables, fruit trees and crops, including apples, grapes, tomatoes, eggplants, and chrysanthemums, each year. Moreover, these pests are vectors for some viruses, including the tomato spotted wilt virus; thus, *F*. *occidentalis* is a key worldwide insect pest of vegetables and other crops.

Few olfactory-related proteins have been characterized in *F*. *occidentalis*, and no CSPs have been reported in Thysanoptera insects to date. An understanding of the molecular mechanisms of the olfactory system and the development of a highly effective strategy for disrupting olfactory-mediated behaviors are critical for facilitating future integrated pest management strategies. The current study aimed to identify and characterize the chemosensory proteins of *F*. *occidentalis*. In this study, we identified, cloned, expressed and purified the first *F*. *occidentalis* chemosensory protein. To further study this CSP, we surveyed its expression in various developmental stages and adult tissues and determined its binding affinities for volatile substances using a competitive-binding fluorescence assay.

## Materials and Methods

### Insect collection and rearing

*F*. *occidentalis* were collected from vegetable greenhouses at the Langfang Experimental Station of the Chinese Academy of Agricultural Sciences in Hebei Province, China. In the laboratory, *F*. *occidentalis* were mass reared, and a laboratory colony was established and maintained at 261°C, 60±5% relative humidity (RH), and a photoperiod of 14: 10 light: dark (L: D).

### RNA isolation and cDNA synthesis

Antennae were excised from 200 anesthetized 1-day-old adults under the microscope, immediately transferred into 1.5 ml eppendorf tubes immersed in liquid nitrogen and stored at -80°C until use. Total RNA for RT-PCR was isolated using TRIzol reagent (Invitrogen, Carlsbad, CA, USA) according to the manufacturer’s protocol and treated with DNase I. The integrity of the total RNA was examined using 1.0% agarose electrophoresis, and the purity was determined from the ratio of A_260_/A_280_ measured by a spectrophotometer. First-strand cDNA was synthesized using M-MLV Reverse Transcriptase according to the manufacturer’s protocol (Promega, Madison, Wisconsin, USA). Single-stranded 3′ RACE-ready and 5′ RACE-ready cDNAs were synthesized from 1 μg of RNA using the 3′ and 5′-full rapid amplification of cDNA end (RACE) core sets (ver.2.0, Takara, Dalian, Liaoning, China), respectively, according to the manufacturer’s instructions.

### Isolation of FoccCSP cDNA

A FoccCSP cDNA fragment was found in the *F*. *occidentalis* transcriptome sequenced at the Beijing Genomics Institute (BGI). We designed forward and reverse primers (Table 1) for PCR amplification of the fragment for sequencing. transcriptome transcriptomeThe amplification conditions were 3 min at 95°C for initial denaturation followed by 30 cycles of 30 s at 94°C, 30 s at 55°C, 1 min at 72°C, and a 10 min final extension at 72°C. Amplified DNA was purified using an AxyPrep DNA gel extraction kit (Axygen), inserted into the *pEASY*-T1 Cloning Vector (TransGen, Beijing, China) and transformed into *E*. *coli Trans*1-T1 competent cells (TransGen, Beijing, China). Positive clones were analyzed by PCR using vector-specific primers and Taq DNA polymerase (Promega). Clones of interest were amplified, and the plasmids were purified and sequenced at Biomad. Three independent clones were sequenced to detect potential PCR mutations.

**Table 1:** PCR Primers.

Purpose/Primer Name	Forward primer (5′-3′)	Reverse primer (5′-3′)
Fragment isolation (RT-PCR)	TGCCTGTTGGCTGTAATC	CCTCTGGACGGGTATCTT
3′RACE Outer Primer	TGTTGGCTGTAATCCTGCTC	TACCGTCGTTCCACTAGTGATTT
3′RACE Inner Primer	AAGTGCTTCTTGGAGGAGGG	CGCGGATCCTCCACTAGTGATTTCACTATAGG
5′RACE Outer Primer	CATGGCTACATGCTGACAGCCTA	GCAGTTGCTGTCAATCACCTCT
5′RACE Inner Primer	CGCGGATCCACAGCCTACTGATGATCAGTCGATG	GAGCAGGATTACAGCCAACA
ORF isolation	gcCCATGGCTGTGGAAAAGTACGAA Underlined *Nco* I restriction enzyme site	cgGGATCCTTACAAAGCAAGAGAAT Underlined *Bam*H I restriction enzyme site
FoccCSP (RT-qPCR)	ATCCTCGGCTCCTTCATCA	CTCTGGACGGGTATCTTTCAC
β-actin (RT-qPCR)	ACGACTTACAACTCCATCA	AGTGCCTCCAGACAAAA

### 3′ RACE-PCR

3′ RACE amplifications were performed using 1 μg 3′ RACE-ready cDNA from *F*. *occidentalis* adult antennae. A gene-specific outer primer was designed based on the fragment sequence obtained above and paired with the 3′ RACE outer primer. The gene-specific inner primer was designed based on the fragment sequence obtained above and paired with the 3′ RACE inner primer. The primers are listed in Table 1. PCRs were performed according to the manufacturer’s instructions for the 3′-full rapid amplification of the cDNA end core set (Takara, Dalian, China). The amplified product was purified from 1.0% agarose gels using an AxyPrep DNA gel extraction kit (Axygen), ligated into the *pEASY*-T1 Cloning Vector (TransGen, Beijing, China) and transformed into *E*. *coli Trans*1-T1 competent cells (TransGen, Beijing, China). Positive clones were analyzed by PCR using vector-specific primers and Taq DNA polymerase (Promega, USA). Clones of interest were amplified, and the plasmids were purified and sequenced at Biomad. Three independent clones were sequenced to detect potential PCR mutations.

### 5′ RACE-PCR

5′ RACE amplifications were performed using 2 μg of 5′ RACE-ready cDNA from adult *F*. *occidentalis* antennae. The gene-specific antisense outer and inner primers for 5′ RACE-PCR were designed from the 3′ RACE results and paired with the 5′ RACE sense outer and inner primers, respectively. The primers are listed in Table 1. PCRs were performed according to the manufacturer’s instructions for the 5′-full rapid amplification of cDNA end core set (Takara, Dalian, China). The purified PCR products were ligated into the *pEASY*-T1 Cloning Vector (TransGen, Beijing, China) and transformed into *E*. *coli Trans*1-T1 competent cells (TransGen, Beijing, China). Positive clones were analyzed and sequenced as described for 3′ RACE-PCR.

### ORF amplification

The full-length cDNA sequence was assembled using the results of 3′ and 5′ RACE, and a fragment from the *F*. *occidentalis* transcriptome by DNAMAN. Primers (Table 1) with NcoI (sense primer) and BamHI (antisense primer) restriction sites was designed to clone the FoccCSP coding region according to the deduced mature amino acid sequence and to trim the putative signal peptide. The amplification conditions were 3 min at 95°C for initial denaturation followed by 30 cycles of 30 s at 94°C, 30 s at 58°C, 1 min at 72°C, and a 10 min final extension at 72°C. The amplified DNA was purified using an AxyPrep DNA gel extraction kit (Axygen), inserted into the *pEASY*-T1 Cloning Vector (TransGen, Beijing, China) and transformed into *E*. *coli Trans*1-T1 competent cells (TransGen, Beijing, China). Positive clones were analyzed by PCR using vector-specific primers. Clones of interest were amplified, and *pEASY*-FoccCSP plasmids were purified and sequenced at Biomad. Three independent clones were sequenced to detect potential PCR mutations.

### Production and purification of recombinant FoccCSP protein

The NcoI—BamHI FoccCSP fragment excised from *pEASY*-FoccCSP was purified using the gel extraction kit (Axygen) and cloned into similarly digested pET-30a (+) vector DNA (Novagen, Darmstadt, Germany), and the recombinant pET-FoccCSP expression plasmid was transformed into *E*. *coli* BL21 (DE3) competent cells. Single colonies were grown overnight in 4 ml of Luria—Bertani broth supplemented with 50 μg/ml kanamycin at 37°C with shaking at 200 rpm. The culture was diluted 1:100 with fresh Luria—Bertani broth (supplemented with 50 μg/ml kanamycin) and grown at 37°C with shaking at 200 rpm until the OD_600_ reached approximately 0.5. Isopropyl β-D-1-thiogalactopyranoside (IPTG) (Merck, Darmstadt, Germany) was then added to the culture to a final concentration of 0.8 mM to induce expression of the target products for approximately 10 h at 28°C. The bacterial cells were then lysed by sonication, and the FoccCSP protein was purified using a Ni-NTA-Sepharose Column (Sangon Biotech, Shanghai, China). Finally, purified His-tagged pET-FoccCSP protein was eluted bysquence nant enterokinasecation. citation is missing. Please ensure that all citations have been inserted prior to submission of cleavage with recombinant enterokinase.

### Western blot analysis

Following electrophoretic separation under denaturing conditions (12.5% sodium dodecyl sulfate polyacrylamide gel electrophoresis (SDS-PAGE)), purified FoccCSP was electroblotted onto a polyvinylidene difluoride (PVDF) membrane using a Bio-Rad Transblot transfer system (Bio-Rad Laboratories, Hercules, CA, USA). Following treatment with blocking solution (40 ml of PBS, 20 μl of Tween-20, 2 g of nonfat dried milk) overnight at 4°C, the membrane was incubated with anti-His tag mouse monoclonal antibody (Cwbio, Beijing, China) (1:2,000 dilution) for 1 h at 37°C, followed by goat anti-mouse IgG (Cwbio, Beijing, China) (1:2,500 dilution) for 2 h at 37°C. Finally, the protein band was visualized using a BCIP/NBT Alkaline Phosphatase Color Development Kit (Cwbio, Beijing, China).

### FoccCSP sequence analysis

FoccCSP-homologs were identified using the NCBI-BLAST network server and aligned using ClustalX1.83 [50]. A phylogenetic tree was constructed using the MEGA 4.0 neighbor-joining method with a p-distance model and pairwise gap deletion. Bootstrapping was performed to estimate the reliability of the branches using 1,000 neighbor-joining replicates. The putative N-terminal signal peptide and its most likely cleavage site were predicted using SignalP V3.0 (http://www.cbs.dtu.dk/services/SignalP/).

### Examination of stage- and tissue-specific FoccCSP expression

Different developmental stages including first and second instar nymphs, pupae, 1-day-old females and males, 5-day-old females and males, 10-day-old females and males, and 15-day-old females and males were immediately frozen in liquid nitrogen and stored at -80°C until use. Various tissues, including antennae, head without antennae, legs, thorax, and adult abdomens, were excised one day following eclosion, immediately frozen in liquid nitrogen, and stored at -80°C until use. Specific primer pairs were designed as shown in Table 1. Total RNA was isolated using TRIzol reagent (Invitrogen) and treated with DNase I (Invitrogen) to remove residual genomic DNA. cDNA was synthesized using a SuperScript III Reverse Transcriptase system (Invitrogen). RNA isolations from each stage and tissue were performed three times. All procedures were performed according to the manufacturer’s instructions, and the resulting cDNA was either used directly or stored at -20°C.

Real-time qPCR was performed using an ABI Prism 7500 Fast Detection System (Applied Biosystems). The *F*. *occidentalis* β-actin gene (GenBank No. GQ290644.1) was used as an endogenous control to normalize target gene expression and correct for sample variation. Amplification reactions were performed in a 20 μl reaction volume containing 10 μl of Go Taq qPCR Master Mix, 2 μl of sample cDNA, 0.5 μl of forward primer, 0.5 μl of reverse primer, and 7 μl of nuclease-free water. The cycling conditions were as follows: 95°C for 120 s and 40 cycles at 95°C for 15 s, followed by 60°C for 60 s. To measure dissociation curves, the PCR products were then heated to 95°C for 1 min, cooled to 55°C for 30 s, heated to 95°C for 30 s and cooled to 60°C for 15 s. No-template controls were included in each experiment. To verify reproducibility, test reactions were performed in triplicate. Relative quantifications were performed using the comparative 2^-ΔΔCt^ method [56–57]. All data were normalized to endogenous β-actin levels of the same stage and tissue samples. The relative fold changes between stages and tissues were calculated using the transcript levels of 10-day-old adult males and abdomens as the baseline for calibration, respectively. Consequently, the relative fold changes in various stages and tissues were assessed by comparing the FoccCSP developmental stage and tissue expression levels with those of 10-day-old adult males and abdomens, respectively.

### Fluorescence-based Ligand Binding Assay

Fluorescence measurements

Fluorescence binding assays were performed using a 970CRT spectrofluorophotometer (Lengguang, Shanghai, China) at 25°C with a 1 cm lightpath quartz cuvette and 10 nm slit width for excitation and emission. FoccCSP protein was diluted from a 2 μM stock solution with 50 mM Tris-HCl buffer (pH 7.4). The fluorescent probe N-phenyl-1-naphthylamine (1-NPN) was diluted from a 1 mM stock solution with methanol. The potential ligands were prepared in a similar manner as 1-NPN.

Intrinsic fluorescence

The intrinsic fluorescence of tryptophan was measured using a 2 μM FoccCSP protein solution. The excitation wavelength was 285 nm, and the emission spectrum was recorded between 290 and 530 nm. Quenching of the intrinsic fluorescence was measured in the presence of 2, 4, 6, 8, 10, 12, 14, 16, 18, and 20 μM 1-NPN under the same conditions.

Binding assays

To measure the affinity of the 1-NPN fluorescent ligand for FoccCSP, the 2 μM protein solution prepared above was titrated with 1 mM 1-NPN to a final concentration between 2 and 20 μM. The affinities of the other potential ligands were measured using 1-NPN as a fluorescent reporter in competitive binding assays using 2 μM solutions of 1-NPN and FoccCSP and the addition of ligands to concentrations of 2–300 μM.

Binding data analysis

To determine the 1-NPN and FoccCSP binding constants, the intensity values corresponding to the maximum fluorescence emissions were plotted against the concentration of free ligand. The bound ligand was evaluated using the fluorescence intensity values assuming that the protein was 100% active and using a saturation stoichiometry of 1:1 protein:ligand. The curves were linearized using the Scatchard Plot program [58]. The dissociation constants for the competitors were calculated from the corresponding IC_50_ values using the equation *K*_*i*_ = [*IC*_50_]/(1+[1-*NPN*]/*K*_1-*NPN*_), where [1-*NPN*] is the free concentration of 1-NPN and *K*_1-*NPN*_ is the dissociation constant of the FoccCSP/1-NPN complex [3, 20].

## Results

### Molecular cloning and cDNA sequencing of FoccCSP

**Fig 1:**
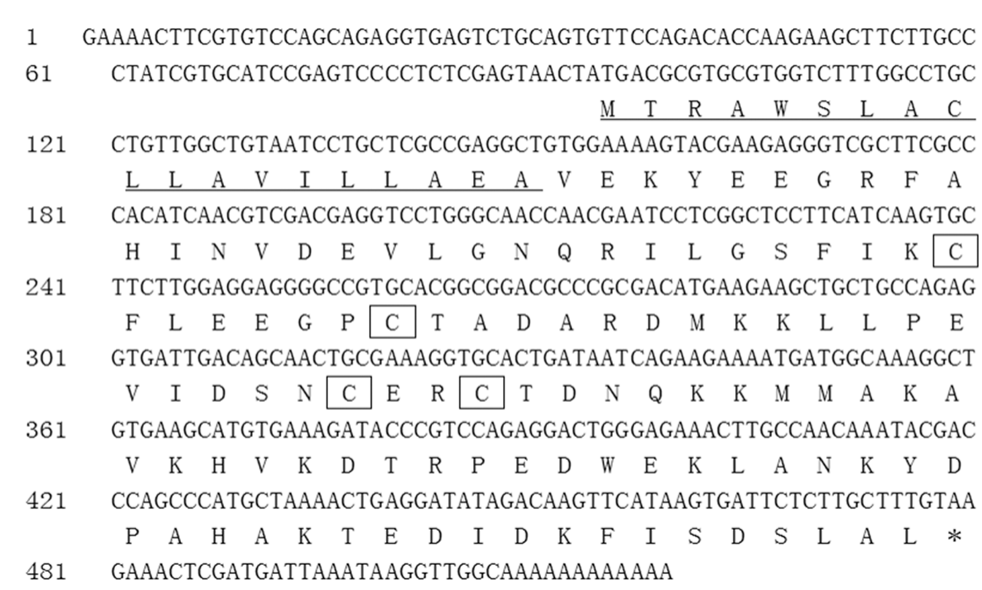
FoccCSP cDNA nucleotide and deduced amino acid sequences. The stop codon is indicated with an asterisk. The putative N-terminal signal peptide is underlined. The four conserved cysteines are boxed.

Full-length cDNA was cloned from *F*. *occidentalis* using RT-PCR and RACE-PCR strategies and then used to deduce an amino acid sequence. According to its high identity with chemosensory proteins from other insects in BLAST searches (http://blast.ncbi.nlm.nih.gov), it was named FoccCSP, and the FoccCSP sequence has been deposited in GenBank (accession number KM035415). This is the first *F*. *occidentalis* chemosensory protein to be identified and cloned. FoccCSP contains a 387 bp open-reading frame encoding a putative 128-amino-acid protein with a molecular weight of 14.51 kDa and an isoelectric point of 5.41. The deduced amino acid sequence contains a 19-amino-acid putative signal peptide at the N-terminus (Fig. 1) and the typical four-cysteine signature of all insect CSPs at conserved positions (C^49^, C^56^, C^75^ and C^78^) when aligned with other known CSPs, conforming to the CSP C_1_-X_6–8_-C_2_-X_16–21_-C_3_-X_2_-C_4_ sequence motif [26].

### FoccCSP homology search in other insect species

**Fig 2:**
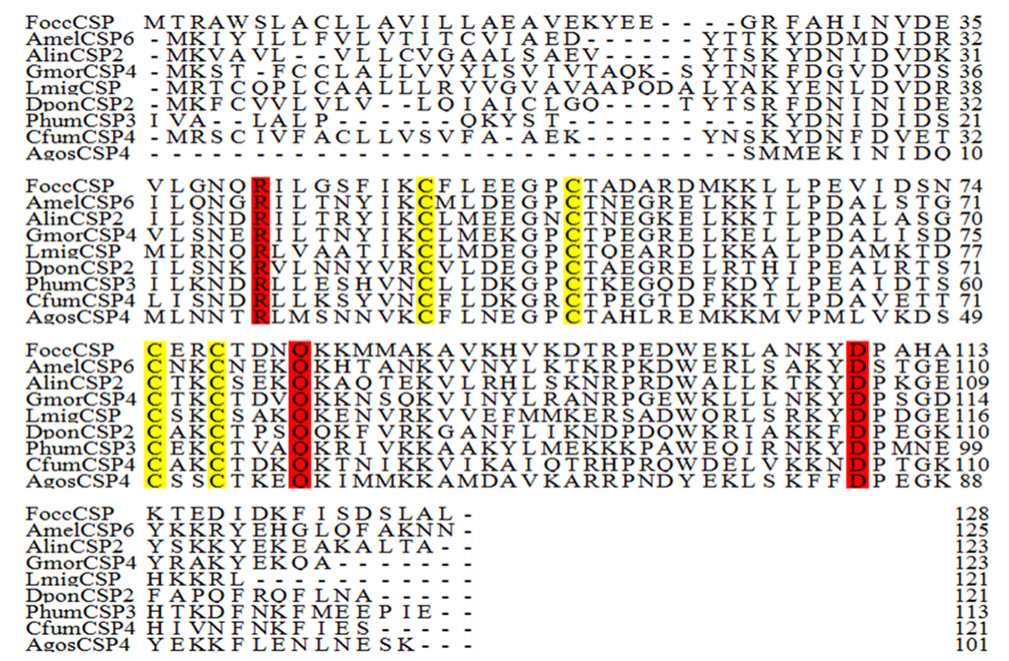
Alignment of FoccCSP with CSPs from other insect orders. The full-length amino acid sequences of FoccCSP homologs were aligned using ClustalX 1.83. The four conserved cysteines are labeled in yellow, and other conserved residues are labeled in red. The insect species, orders and GenBank accession numbers are as follows: FoccCSP: *Frankliniella occidentalis*, Thysanoptera, KM035415; AmelCSP6: *Apis mellifera*, Hymenoptera, NP_001071287.1; AlinCSP2: *Adelphocoris lineolatus*, Hemiptera, ACZ58021.1; GmorCSP4: *Glossina morsitans morsitans*, Diptera, CBA11330.1; LmigCSP: *Locusta migratoria*, Orthoptera, CAJ01472.1; DponCSP2: *Dendroctonus ponderosae*, Coleoptera, AGI05172.1; and PhumCSP3: *Pediculus humanus corporis*, Anoplura, XP_002432597.1.

We searched for FoccCSP homologs in other insects using BLASTX with an e-value cut-off of 10e-5 [9, 59–60]. The results of this search revealed 99 insect CSPs with homology to FoccCSP (Fig. 2 and S1 Table). Among them there are 36 Dipteran CSPs, 22 Hymenopteran CSPs, 20 Lepidopteran CSPs, 12 Homopteran CSPs, 3 Coleopteran CSPs, 3 Orthopteran CSPs, 2 Hemipteran CSPs and 1 Anopluran CSP despite the fact that FoccCSP is from *F*. *occidentalis*, a Thysanopteran. Therefore, FoccCSP is from a different order of insect than other CSPs, and the homology between FoccCSP and these 99 insect CSPs may be relatively low.

### Phylogenetic analysis

**Fig 3:**
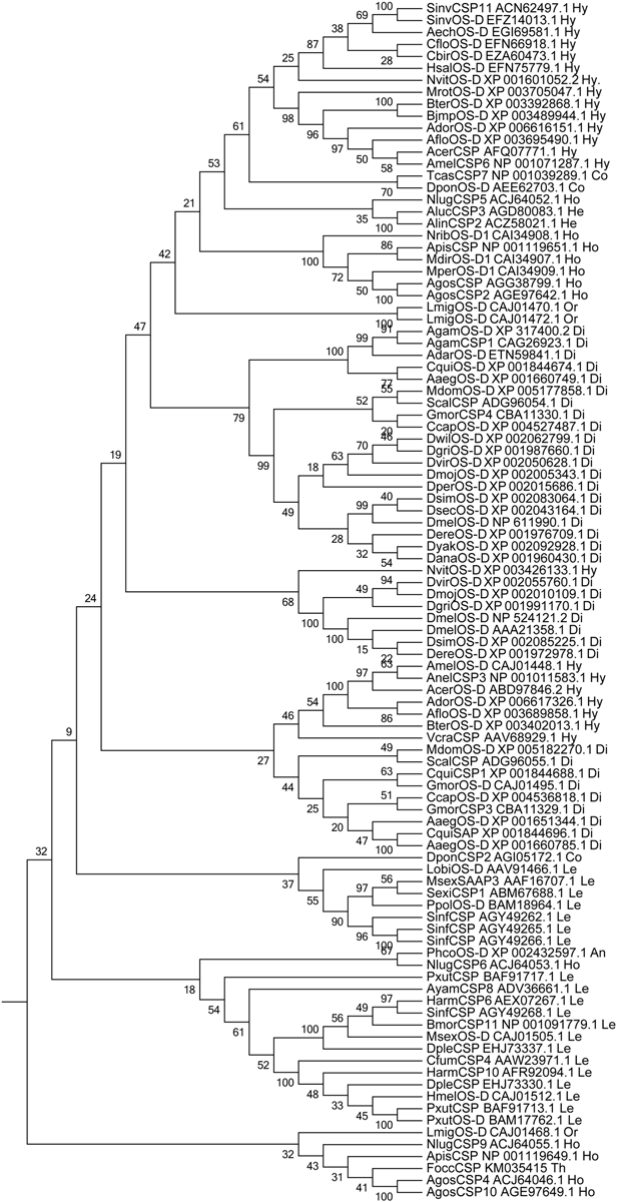
Similarity tree of FoccCSP protein compared with other insect CSPs. Neighbor-joining tree of sequences similar to FoccCSP were chosen based on their BLASTP e-value scores. Bootstrap values (%) are based on 1,000 replicates. Protein accession numbers and orders are included with the species abbreviations for taxon identification. Order abbreviations: Di (Diptera), Hy (Hymenoptera), Le (Lepidoptera), Ho (Homoptera), Co (Coleoptera), Or (Orthoptera), He (Hemiptera), An (Anoplura), Th (Thysanoptera). Species abbreviations: Aaeg (*Aedes aegypti*), Ador (*Apis dorsata*), Aflo (*Apis florea*), Amel (*Apis mellifera*), Bmor (*Bombyx mori*), Gmor (*Glossina morsitans morsitans*), Lobl (*Lonomia obliqua*), Nvit (*Nasonia vitripennis*), Sinf (*Sesamia inferens*), Sinv (*Solenopsis invicta*), Aech (*Acromyrmex echinatior*), Apis (*Acyrthosiphon pisum*), Alin (*Adelphocoris lineolatus*), Aaeg (*Aedes aegypti*), Adar (*Anopheles darlingi*), Agam (*Anopheles gambiae str*. *PEST*), Agam (*Anopheles gambiae*), Ayam (*Antheraea yamamai*), Agos (*Aphis gossypii*), Acer (*Apis cerana cerana*), Ador (*Apis dorsata*), Aflo (*Apis florea*), Amel (*Apis mellifera*), Aluc (*Apolygus lucorum*), Bimp (*Bombus impatiens*), Bter (*Bombus terrestris*), Cflo (*Camponotus floridanus*), Cbir (*Cerapachys biroi*), Ccap (*Ceratitis capitata*), Cfum (*Choristoneura fumiferana*), Cqui (*Culex quinquefasciatus*), Dple (*Danaus plexippus*), Dpon (*Dendroctonus ponderosae*), Dana (*Drosophila ananassae*), Dere (*Drosophila erecta*), Dgri (*Drosophila grimshawi*), Dmel (*Drosophila melanogaster*), Dmoj (*Drosophila mojavensis*), Dper (*Drosophila persimilis*), Dsec (*Drosophila sechellia*), Dsim (*Drosophila simulans*), Dvir (*Drosophila virilis*), Dwil (*Drosophila willistoni*), Dyak (*Drosophila yakuba*), Focc (*Frankliniella occidentalis*), Gmor (*Glossina morsitans*), Hsal (*Harpegnathos saltator*), Hmel (*Heliconius melpomene*), Harm (*Helicoverpa armigera*), Lmig (*Locusta migratoria*), Msex (*Manduca sexta*), Mrot (*Megachile rotundata*), Mdir (*Metopolophium dirhodum*), Mdom (*Musca domestica*), Mper (*Myzus persicae*), Nvit (*Nasonia vitripennis*), Nrib (*Nasonovia ribisnigri*), Nlug (*Nilaparvata lugens*), Ppol (*Papilio polytes*), Pxut (*Papilio xuthus*), Phum (*Pediculus humanus corporis*), Sinf (*Sesamia inferens*), Sinv (*Solenopsis invicta*), Sexi (*Spodoptera exigua*), Scal (*Stomoxys calcitrans*), Tcas (*Tribolium castaneum*), Vcra (*Vespa crabro*).

The CSP-related sequences including FoccCSP were aligned in ClustalX1.83 (data not shown). An unrooted neighbor-joining tree was constructed to display the hypothesized relationships among these CSP-related proteins. The alignment analysis showed that apart from the four conserved cysteines contained in all CSPs, an arginine residue at the eighth amino acid before the first conserved cysteine and a glutamine residue at the fourth position following the final conserved cysteine are also conserved in these sequences. FoccCSP showed 31–50% sequence identity with other insect CSPs. FoccCSP showed relatively high identity with *A*. *gossypii* CSP4 (50%, AgosCSP4, GenBank accession ACJ64046.1) and CSP10 (45%, AgosCSP10, GenBank accession AGE97649.1), indicating that FoccCSP has relatively high homology with *A*. *gossypii* CSPs. FoccCSP showed 44% identity with *D*. *mojavensis* OS-D (DmojOS-D, GenBank accession XP_002005343.1), *M*. *rotundata* OS-D (MrotOS-D, GenBank accession XP_003705047.1), *A*. *lucorum* CSP3 (AlucCSP3, GenBank accession AGD80083.1) and *L*. *migratoria* OS-D (LmigOS-D, GenBank accession CAJ01470.1). FoccCSP has low identity with the following CSPs: 35% identity with *Choristoneura fumiferana* CSP4 (CfumCSP4, GenBank accession AAW23971.1), *Manduca sexta* SAP3 (MsexSAP3, GenBank accession AAF16707.1), *Aedes aegypti* OS-D (AaegOS-D, GenBank accession XP_001651344.1) and *Culex quinquefasciatus* CSP1 (CquiCSP1, GenBank accession XP_001844688.1); 34% identity with *Tribolium castaneum* CSP7 (TcasCSP7 GenBank accession NP_001039289.1) and *Nilaparvata lugens* CSP6 (NlugCSP6, GenBank accession ACJ64053.1); 33% identity with *Aedes aegypti* OS-D (AaegOS-D, GenBank accession XP_001660785.1); and 32% identity with *Dendroctonus ponderosae* CSP2 (DponCSP2, GenBank accession AGI05172.1). At 30% identity, FoccCSP has the lowest identity with *Antheraea yamamai* CSP8 (AyamCSP8, GenBank accession ADV36661.1). The sequence similarity tree using a neighbor-joining tree (Fig. 3) shows that these 100 CSPs are subdivided into two main groups. FoccCSP is in one group with AgosCSP4 (GenBank accession ACJ64046.1), AgosCSP10 (GenBank accession AGE97649.1), ApisCSP (GenBank accession NP 001119649.1) and NlugCSP9 (GenBank accession ACJ64055.1), which are all from homopteran insects, suggesting that these genes may have developed from a common ancestral gene.

### FoccCSP stage and tissue distribution

**Fig 4:**
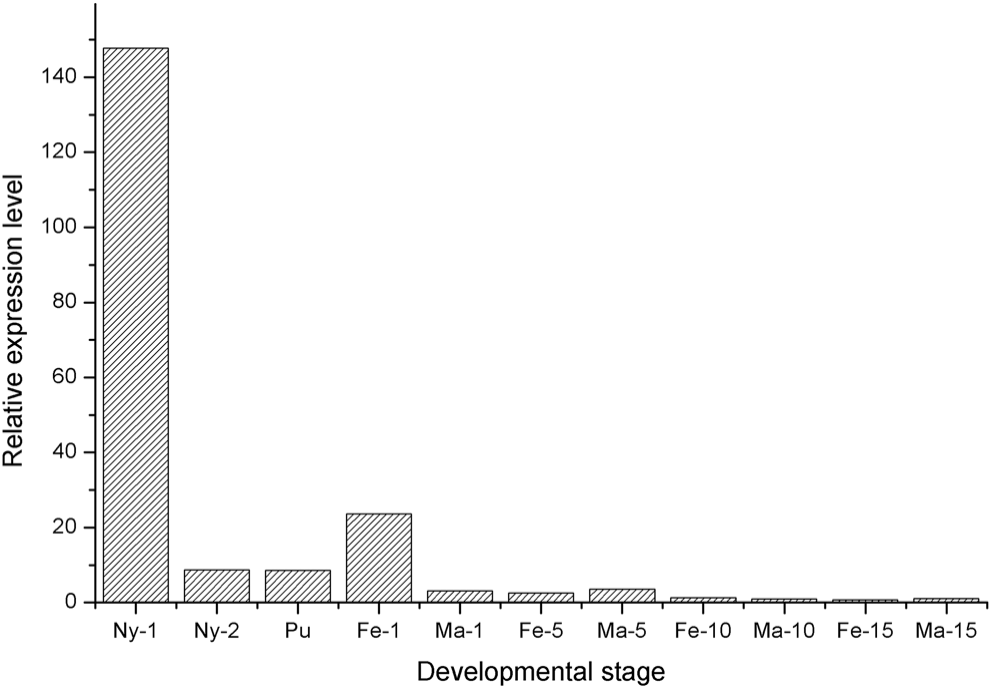
Relative expression levels of FoccCSP in various *F*. *occidentalis* developmental stages.

**Fig 5:**
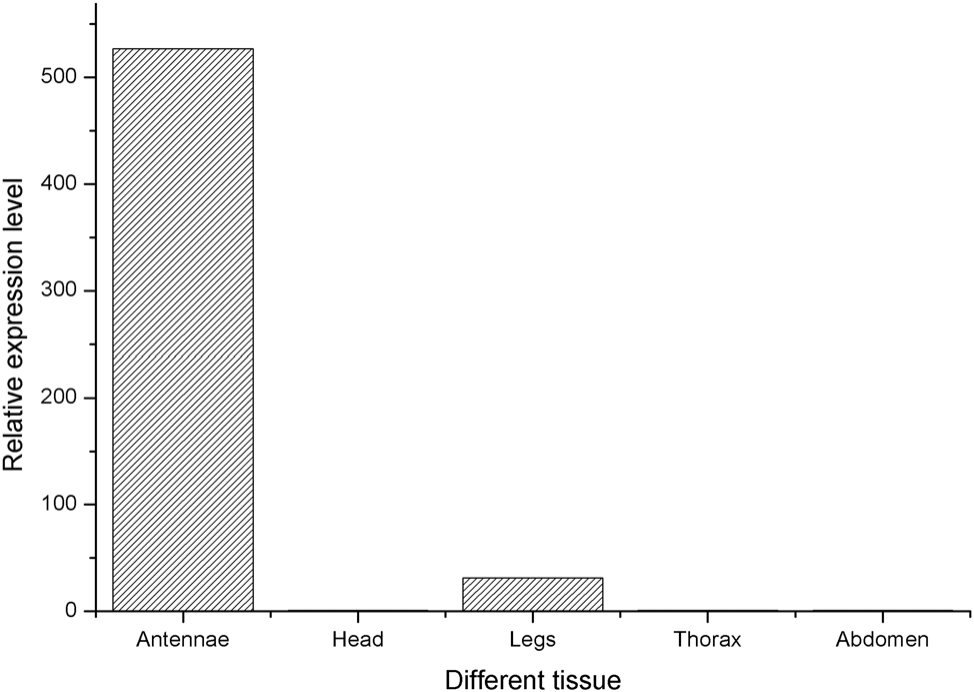
Relative expression levels of FoccCSP in various adult tissues one day after eclosion.

To further study the function of FoccCSP, we examined its expression profile using real-time qPCR and the comparative 2^-ΔΔCt^ method to precisely compare relative FoccCSP transcript levels from different stages and tissues (Figs. 4 and 5).

To study the tissue distribution of FoccCSP, abdominal FoccCSP expression was used for calibration. FoccCSP was expressed in all tissues but was predominantly expressed in antennae. FoccCSP transcript levels were 526.91 times greater in the antennae than in the abdomen, followed by leg tissue at 31.29 times greater than in the abdomen. Low FoccCSP transcript levels were detected in in the thorax and head (only 0.99 and 0.78 times the expression of the abdomen, respectively).

To study the distribution of FoccCSP across developmental stages, FoccCSP expression in 10-day-old adult males was used for calibration. FoccCSP expression levels varied throughout development. First-instar nymphs had the highest expression at 147.72 times greater than that observed for 10-day-old adult males, followed by 1-day-old adult females. FoccCSP transcript levels in second-instar nymphs, pupae, 5-day-old adult males, 1-day-old adult males, 5-day-old adult females, 10-day-old adult females and 15-day-old adult males were progressively reduced at 8.71, 8.59, 3.56, 3.15, 2.57, 1.28 and 1.12 times the expression 10-day-old adult males, respectively. The lowest FoccCSP transcript levels were observed for 15-day-old adult females, which had only 0.69 times the expression of 10-day-old adult males.

### Fluorescence binding assays

**Fig 6:**
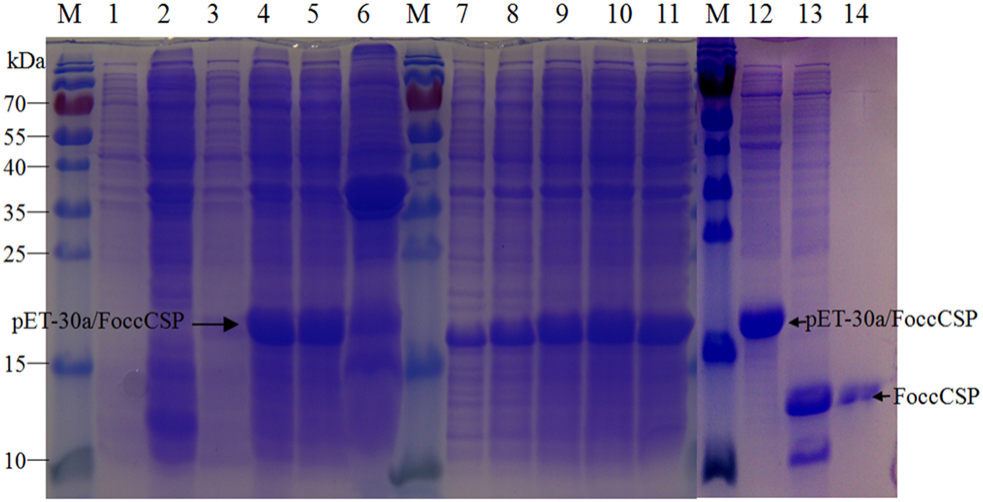
Verification of FoccCSP protein expression and purification by SDS-PAGE. Lane 1: uninduced BL21 bacteria with pET-30a (+) vector, Lane 2: induced BL21 bacteria with pET-30a (+) vector, Lane 3: uninduced BL21 bacteria with pET-30a/FoccCSP vector, Lane 4: induced BL21 bacteria with pET-30a/FoccCSP vector, Lane 5: supernatant of induced BL21 bacteria with pET-30a/FoccCSP vector, Lane 6: sediment of induced BL21 bacteria with pET-30a/FoccCSP vector. Lanes 7–11: different concentrations of IPTG used to induce recombinant protein (2, 4, 6, 8, 10 mM from lanes 7 to 11). Lane 12: protein before cleavage with recombinant enterokinase, Lane 13: protein cleaved by recombinant enterokinase, Lane 14: purified protein. Protein molecular weight marker (M), from the top: 170, 130, 100, 70, 55, 40, 35, 25, 15, and 10 kDa.

To obtain purified FoccCSP and measure FoccCSP protein binding affinities for volatile substances, we expressed the FoccCSP gene in a bacterial system and induced mass production using isopropyl β-D-1-thiogalactopyranoside. We then purified the recombinant protein by affinity chromatography using a Ni-NTA-Sepharose column and gel filtration. The size and purity of the recombinant protein were examined using SDS-PAGE (Fig. 6).

**Fig 7:**
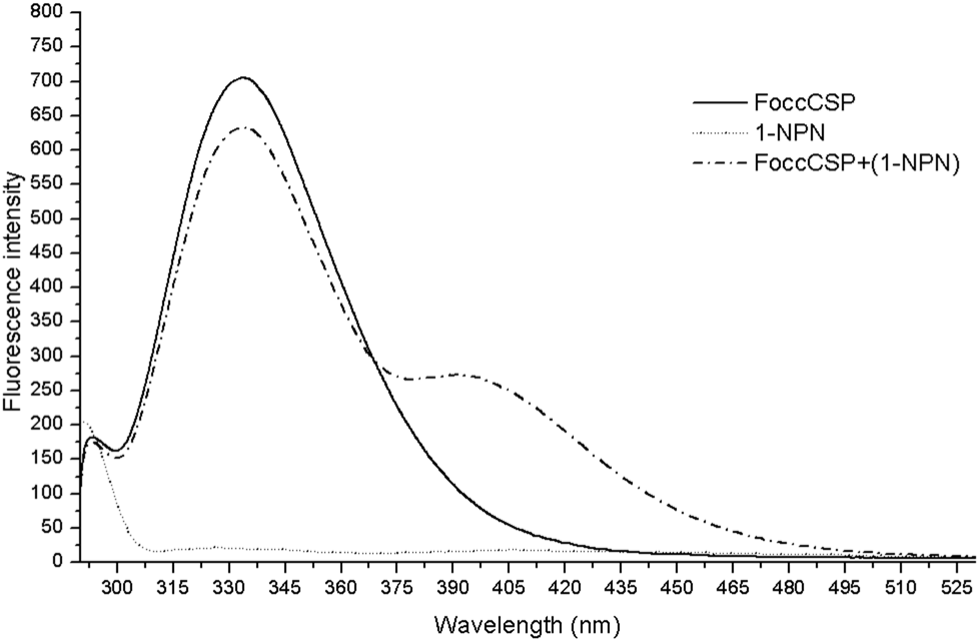
FoccCSP and 1-NPN fluorescence spectra. FoccCSP: FoccCSP alone (2 μM); 1-NPN: 1-NPN alone (2 μM); FoccCSP+(1-NPN): FoccCSP and 1-NPN (2 μM each)．

The fluorescence displacement assays were performed using the fluorescent probe N-phenyl-1-naphthylamine (1-NPN) as a reporter. When excited with 285 nm light, 1-NPN produced a weak fluorescent peak with a maximum at 405 nm. When the 1-NPN probe was bound to FoccCSP protein (2 μM) and excited with 285 nm light, the fluorescence emission peak shifted from 405 to 399 nm and significantly increased in intensity (Fig. 7).

**Fig 8:**
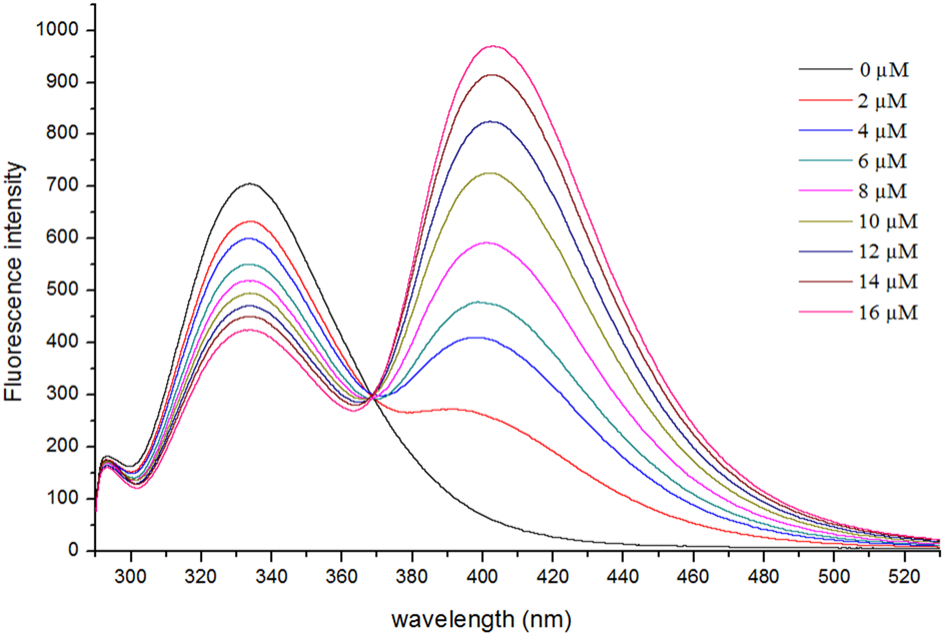
Effects of 1-NPN on FoccCSP fluorescence.

**Fig 9:**
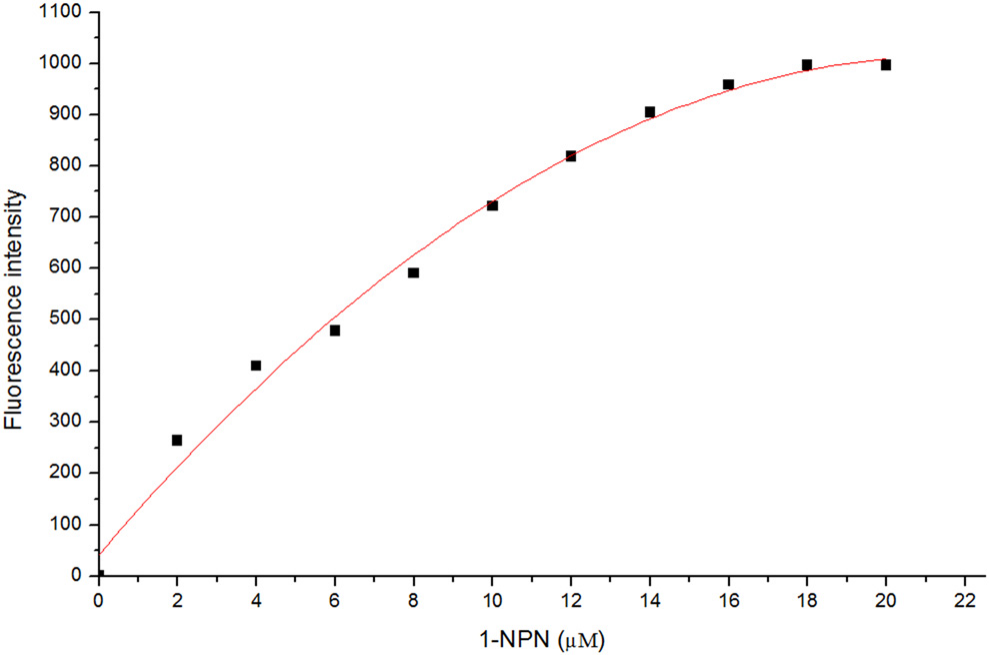
Binding curves and Scatchard plots of 1-NPN binding to FoccCSP protein.

Mature FoccCSP protein contains a single tryptophan at position 82. To verify that 1-NPN was bound inside the protein rather than interacting with its surface, we measured the intrinsic tryptophan fluorescence quenching of the protein and the binding affinity of 1-NPN to FoccCSP. When excited with 285 nm light, the FoccCSP tryptophan residue exhibited an emission maximum at 333 nm, indicating that tryptophan is located within the FoccCSP protein core in a rather hydrophobic environment [3, 20]. Upon the addition of 1-NPN, the intrinsic fluorescence was quenched by 1-NPN in a dose-dependent manner between 2 and 16 μM (Fig. 8), indicating that 1-NPN was bound within FoccCSP. Assuming that the concentration of bound 1-NPN equals the concentration of the protein under saturating conditions, we can use the fluorescence intensity at 333 nm to calculate the concentration of bound 1-NPN. The binding curves and relative Scatchard plots are shown in Fig. 9. The FoccCSP/1-NPN complex dissociation constant was 11.81 μM.

Ligand-binding experiments

Using 1-NPN as a fluorescent reporter and 19 ligands as competitors, the FoccCSP binding affinities for 19 ligands were determined and are listed in Table 2. These ligands are all volatile substances released by *F*. *occidentalis* host plants, e.g., cucumber, tomato, and eggplant. With the exception of salicylaldehyde, the ligands effectively displaced the fluorescent 1-NPN probe from the complex and decreased the fluorescence intensity of FoccCSP/1-NPN to 50% of the initial value. FoccCSP binds strongly to anisic aldehyde (released by *Foeniculum vulgare* and *Illicium verum*), with a K_D_ of 10.50 μM. FoccCSP also exhibited high-affinity binding to geraniol (released by *Allium sativum L*. [garlic]) and methyl salicylate (released by *Solanum lycopersicum* and *Cucumis sativus Linn*), with K_D_ values of 15.35 and 35.24 μM, respectively. FoccCSP showed medium-affinity binding to ethyl nicotinate, methyl isonicotinate, benzaldehyde, ethyl benzene, citronellol, citronellal, pelargonic aldehyde, linalool, eucalyptol, hexane and eugenol, having K_D_ values between 47.93 and 88.87 μM. Among them, FoccCSP showed relatively weak-affinity binding to benzyl acetate, neroli, 3-phenyl aldehyde and benzyl alcohol, having K_D_ values of 90.54, 91.16, 99.73 and 109.43 μM, respectively.

**Table 2:** Competitive binding of candidate ligands with 1-NPN and recombinant FoccCSP protein.

ligands	IC50 (μM)	Dissociation constant (μM)	ligands	IC50 (μM)	Dissociation constant(μM)
Anisic aldehyde	11.3944	10.5047	Linalool	85.6408	78.9534
Geraniol	15.3463	14.1480	Eucalyptol	92.3824	85.1686
Methyl salicylate	35.2426	32.4906	Hexane	94.2109	86.8544
Ethyl nicotinate	51.9911	47.9313	Eugenol	96.3941	88.8671
Methyl isonicotinate	51.9914	47.9316	Benzyl acetate	98.2135	90.5444
Benzaldehyde	52.4622	48.3657	Neroli	98.8865	91.1648
Ethyl benzene	63.4222	58.4698	3- phenyl aldehyde	108.1812	99.7338
Citronellol	75.7733	69.8565	Benzyl alcohol	118.6962	109.4277
Citronellal	76.0562	70.1173	Salicylaldehyde	#NUM!	#NUM!
Pelargonic aldehyde	79.0009	72.8320			

## Discussion

As described above, a large number of CSPs have been identified in some insect orders, e.g., Lepidoptera, Hymenoptera, Blattaria, Phasmatodea, Orthoptera, Diptera, and Hemiptera; however, no CSPs have been identified and reported in Thysanoptera to date.

In this study, we identified and cloned a chemosensory protein (FoccCSP) from the western flower thrips *Frankliniella occidentalis* (Thysanoptera: Thripidae). This is the first CSP identified in *F*. *occidentalis* and even in Thysanoptera. FoccCSP was named as such for two reasons: first, it shares high identity with CSPs from other insects by BLASTP, and second, FoccCSP possesses the CSP common signatures of low molecular mass, an isoelectric point between 5 and 6 [61], and four conserved cysteine residues that conform to the CSP common cysteine sequence spacing pattern [26].

The BLASTX homology search result revealed a large number of insect CSPs. Most are from Diptera, Hymenoptera and Lepidoptera, whereas a few are from Homoptera, Coleoptera, Orthoptera, Hemiptera and Anoplura. These orders have FoccCSP homologs with e-values of less than 10e-20, indicating considerable support, meaning that these sequences absolutely have homology with FoccCSP.

Notably, using CLUSTALX1.83 alignments, we identified an arginine residue and glutamine residue that are also completely conserved between the examined sequences and FoccCSP, in addition to the four conserved cysteines. This finding was not previously reported and may be key to the homology between FoccCSP and the examined CSPs. The alignment of FoccCSP with these CSPs showed only 31–50% sequence identity, contrary to the opinion that CSPs are highly conserved, as they share approximately 50% sequence identity even between CSPs from different orders [15, 18, 45]. The primary cause of the low sequence identity is likely that FoccCSP comes from *F*. *occidentalis*, which is part of the order Thysanoptera, whereas the other CSPs are from other orders. This reduced homology also suggests that either Thysanoptera CSPs are distantly related to CSPs from other orders or that FoccCSP has evolved rapidly.

The relationship hypothesized by the phylogenetic analysis produced using MEGA6 software indicates that FoccCSP is in a large group with only 5 other CSPs, among which 4 CSPs are from Homopteran insects (AgosCSP4, AgosCSP10, ApisCSP and NlugCSP9), suggesting that these 6 genes have likely diversified via gene duplication of a common ancestral gene. The other 96 CSPs are in a separate group that is likely to provide further evidence that the evolutionary direction of FoccCSP differs significantly from that of other CSPs.

Varied expression indicates that this gene may have different functions in some tissues or developmental stages. In this report, we used real-time qPCR to examine the temporal and spatial patterns of FoccCSP expression. The analysis of transcript levels in different tissues showed that FoccCSP is expressed in all of the tissues described in this paper, indicating that FoccCSP has a broad tissue expression profile in *F*. *occidentalis*. This result is consistent with the view that CSPs are broadly distributed throughout the body in various tissues, such as the antennae, head, legs, thorax, ejaculatory duct, and pheromone glands [45]. In contrast, some CSPs have been reported to be specifically expressed in the antennae [37, 62]. FoccCSP transcript levels are varied in different tissues. FoccCSP expression is principally enriched in antennae and leg tissue. There are many olfactory sensilla and chemosensory organs in antennae and legs; thus, it is likely that FoccCSP may be involved in recognizing and transporting semiochemicals or some hydrophobic molecules from the lymph to chemosensory receptors.

The analysis of FoccCSP expression throughout development showed the highest transcript levels in first instar nymphs, suggesting that FoccCSP may play a crucial role in first-instar nymph development or in life activities that are particular to that stage. This result is consistent with the opinion that CSPs are involved in the development of specific stages or tissues in insects, such as enhancing p10 expression during *Periplaneta americana* leg regeneration [39, 48]. Furthermore, the transcript levels of different adult stages revealed that the transcript is relatively highly expressed in 1-day-old adult females and then gradually decreases with age, indicating that FoccCSP may have a role in some adult behaviors and its function might decrease with increased adult age.

All 19 competitor ligands used in our fluorescence binding assay are volatile substances that are released by *F*. *occidentalis* host plants, e.g., cucumber, tomato, and eggplant. Furthermore, each volatile substance could clearly attract *F*. *occidentalis* in earlier experiments [63]. In the current study, we found that only salicylaldehyde was unable to displace the fluorescent 1-NPN probe from the FoccCSP/1-NPN complex to reduce the fluorescence intensity to 50% of the initial value, indicating that FoccCSP nearly does not bind salicylaldehyde. In contrast, FoccCSP can strongly bind anisic aldehyde, geraniol and methyl salicylate, which are primarily extracted from *Foeniculum vulgare*, *Illicium verum*, *Allium sativum L*. (Garlic), *Solanum lycopersicum* and *Cucumis sativus Linn*, indicating that FoccCSP is important for *F*. *occidentalis* recognition, seeking, location of and feeding on these host plants. FoccCSP showed medium binding to ethyl nicotinate, methyl isonicotinate, benzaldehyde, ethyl benzene, citronellol, citronellal, pelargonic aldehyde, linalool, eucalyptol, hexane and eugenol, indicating that FoccCSP, in addition to other CSPs or odorant binding proteins (OBPs) of *F*. *occidentalis*, likely participates in the recognition of, transport of, or other physiological functions pertaining to these chemicals.

In summary, FoccCSP was expressed in *E*. *coli* and purified by affinity chromatography using a Ni-NTA-Sepharose Column. Our expression pattern data and fluorescence binding assays suggest that FoccCSP may be involved in recognizing, binding and transporting semiochemicals or in regulating specific developmental processes of the first instar nymph; however, the exact physiological roles of FoccCSP remain unclear. Additional studies are needed to better understand the possible functions of the CSP family in *F*. *occidentalis*. Our research on FoccCSP serves as a useful starting point for the identification of additional olfactory-related genes and study of the olfactory system in *F*. *occidentalis* and even in Thysanoptera, as well as for designing new and effective strategies to control this insect pest in the future.

## Supporting Information

S1 Table Alignment of FoccCSP with other CSPs. (XLS)
